# Sequential Treatment of Arsenic Trioxide Followed by All Trans Retinoic Acid with Anthracyclines has Excellent Long-Term Cure in Acute Promyelocytic Leukemia

**DOI:** 10.1007/s12288-020-01311-x

**Published:** 2020-06-26

**Authors:** Santhosh Kumar Devadas, Hasmukh Jain, Bausaheb Bagal, Manju Sengar, Uma Dangi, Navin Khattry, Pratibha Amre, Nikhil Patkar, P. G. Subramaniam, Reena Nair, Hari Menon

**Affiliations:** 1 Department of Medical Oncology, Ramaiah Medical College and Hospital, Bengaluru, India; 2 Department of Medical Oncology, Tata Memorial Center, Lower Parel, Mumbai, India; 3 Department of Cytogenetics, Tata Memorial Center, Mumbai, India; 4 Department of Hemato-pathology, Tata Memorial Center, Mumbai, India

**Keywords:** APL, Long term cure, Sequential treatment, Early mortality

## Abstract

Acute promyelocytic leukemia (APL) remains the most curable myeloid leukemia made feasible through effective use of two differentiating agents, all trans retinoic acid (ATRA) and arsenic trioxide (ATO) with or without chemotherapy (CT). However, early morbidity and mortality remains a problem. With the objective of reducing early death a strategy of sequential induction ATO followed by consolidation ATRA in combination with CT was adopted by our group. The long-term outcomes of patient of APL treated on this sequential approach at our center was analyzed. In this retrospective analysis of prospectively maintained database consecutive adult patients with APL irrespective of their Sanz risk group were treated using a protocol of ATO (10 mg IV infusion over 3 h daily for 45 days) in the first phase followed by ATRA (45 mg/m^2^ for 60 days) in combination with Daunorubicin (60 mg/m^2^ for 3 days × 3 cycles) in second phase. All patients received maintenance ATRA (45 m/m^2^ for 15 days every 3 months) for a period of 18 months in phase 3. Patients were monitored for cytogenetic and molecular responses after phase 1 and 2. All patients were followed up for toxicity, event free and overall survival. 131 consecutive patients were treated in this study. At a median follow up of 60 months, 84.81% patients are alive with an overall event free survival (EFS) of 77.82%. Sanz low risk patients fared better (85%) versus intermediate and high-risk patients who had a 76% EFS. Proportion of patients alive at last follow up were 100% in Sanz low risk group and 82% in intermediate and high-risk group. The sequential schedule showed excellent tolerance and toxicity profile when treating newly diagnosed APL. The long-term follow-up data shows comparable if not better survival compared with the published real-world data and this has been consistent across all risk group.

## Introduction

Acute promyelocytic leukemia (APL) is a unique subset of acute myeloid leukemia (AML) whose pathogenesis revolves around a translocation involving the retinoic acid receptor (RAR) locus on chromosome 17, most commonly the t (15; 17) (q22; q11), giving rise to the *PML*–*RARα* fusion gene that encodes an oncoprotein. This oncoprotein disrupts the physiological interaction of the retinoic acid with RARα causing the latter to be converted to a transcription activator. This leads to maturation arrest at the promyelocytic stage which in turn ends in the manifestation of the disease characterized by myeloproliferation and bleeding diathesis [1]. While it is well known that APL is exquisitely sensitive to chemotherapy, the catastrophic intrinsic bleeding manifestation further aggravated by chemotherapy has resulted in significant early mortality with the use of chemotherapy alone. The use of ATRA was a paradigm shift in the management of APL and the molecule induced leukemic cell differentiation leading to remission of the disease with decline in early treatment related mortality. Its single agent use however did not yield as good durable remissions. The combination of ATRA with chemotherapy subsequently became the standard of treatment with excellent long-term outcomes. However, early death still was a problem [2]. Arsenic trioxide (ATO) is a very potent agent for use in APL and is capable of inducing and maintaining long term remissions when used as a single agent especially in low risk disease [3]. More recently the combination of ATRA with ATO has shown that comparable results can be achieved in patient with low and intermediate risk group as defined by the Sanz criteria. Therefore, many investigators believe that chemotherapy need not be used in therapy of APL [4, 5]. However, our premise was that the best therapeutic agents have to be utilized in the frontline setting itself to optimize outcomes while reducing early mortality. Since only ATO when used alone as induction without chemotherapy may reduce early mortality, we embarked on a strategy to use of all three active agents in a sequential manner wherein ATO was used as an agent to induce remissions initially followed by standard ATRA with chemotherapy in consolidation with a primary objective to reduce mortality while optimizing outcomes. The long-term outcomes of this strategy are presented and discussed in this report.

## Materials and Methods

### Patients

131 continuous patients of APL treated at the adult hematology division of Tata Memorial Hospital, Mumbai, India between January 2008 and December 2012 were included in this retrospective analysis. This study was approved by the institutional ethics Committee and consent was waivered since it is a retrospective study. Eligibility criteria included a morphological diagnosis of AML M3, according to FAB criteria and cytogenetic demonstration of t (15; 17) and/or demonstration of *PML*–*RAR* transcript. Genetic variations of APL other than (*PML; RARA*) were excluded from the study.

### Protocol

Included a strategy of induction followed by consolidation therapy/post remission therapy followed by maintenance therapy. Induction consisted of treatment with Injection Arsenic trioxide at a maximum dose of 0.15 mg/kg/day for 45 days. After documentation of remission by morphology, cytogenetic assessment and molecular detection of transcript, patients received consolidation with ATRA, 45 mg/m^2^/day orally for 60 days continuously and Daunorubicin, 60 mg/m^2^ for 3 days × 3 such cycles in the initial patients which was reduced to 45 mg/m^2^ for 3 days for 3 cycles subsequently for some patients due to concerns regarding possible cardiotoxicity. Consolidation was started as soon as morphological remission was documented and the counts were normal and patient was clinically stable with acceptable organ function. Each cycle of daunorubicin consolidation was started soon after count recovery from cytopenia of the previous cycle. Following consolidation maintenance was started with ATRA 45 mg/m^2^ for 15 days every 3 months × 6 such cycles. Following this patient was kept on follow up every 4 months. In case patient did have persistence of PCR positivity, they were offered additional therapy with high dose cytarabine followed by maintenance with or without 6-mercaptopurine and methotrexate. RQ-PCR monitoring of the PML–RAR transcript was done at the end of induction from bone marrow sample and if positive after induction it was repeated after each consolidation till the transcript became negative.

### Supportive Care

Patients were admitted for the initiation of ATO. They were monitored closely during induction for signs and symptoms of infection, bleeding, and differentiation syndrome. All patients underwent twice daily monitoring of complete blood count (CBC), coagulation, fibrinogen and serum electrolytes till they were clinically stable and then once daily while admitted in the hospital. Regular monitoring of liver function and renal function tests along with calcium, magnesium, phosphorus levels were done during induction phase. ECG was done twice weekly while the patient was on ATO to monitor QTc interval. Following patient’s discharge, they were seen at least twice weekly while on ATO with monitoring of their CBC, coagulation, biochemical profile and ECG. Antibiotics/antifungals were used when indicated if there was evidence of infection. Fibrinogen level was maintained above 200 mg/dl with cryoprecipitate support as and when required. Platelet count maintained above 50,000/mm^3^ during induction with ATO. If electrolytes imbalances were corrected accordingly. If the QTc interval was more than 0.5 ms arsenic was withheld temporarily. If the patient developed clinical signs or symptoms of differentiation syndrome Injection Dexamethasone at a dose of IV 8 mg twice daily was initiated and not lasting more than 3 days. High counts during initial phase and high leucocyte counts were controlled by instituting Hydroxyurea.

### Response Assessment

Following completion of induction treatment responses were assessed by bone marrow assessment. The *PML RAR* transcript level was assessed and RQ-PCR negativity was the aim of therapy. If marrow positive for *PML: RAR* transcript post induction it was repeated after consolidation. If RQ PCR was positive after consolidation too further treatment was provided in the form of high dose cytarabine for 2 or 3 cycles. The overall survival was calculated from the time from diagnosis until death from any cause. Event-free survival was the time from diagnosis to any treatment failure, including disease progression, discontinuation of treatment for any reason (e.g. toxicity, patient preference, initiation of new treatment) or death.

### Statistical Methods

Survival analysis was done with Kaplan Meir analysis. The OS and EFS were compared with Log rank test. All survival analysis is reported as ± 1 SE.P. Statistical analysis was done with SPSS version 21 (IBM.USA).

## Results

**Fig. 1:**
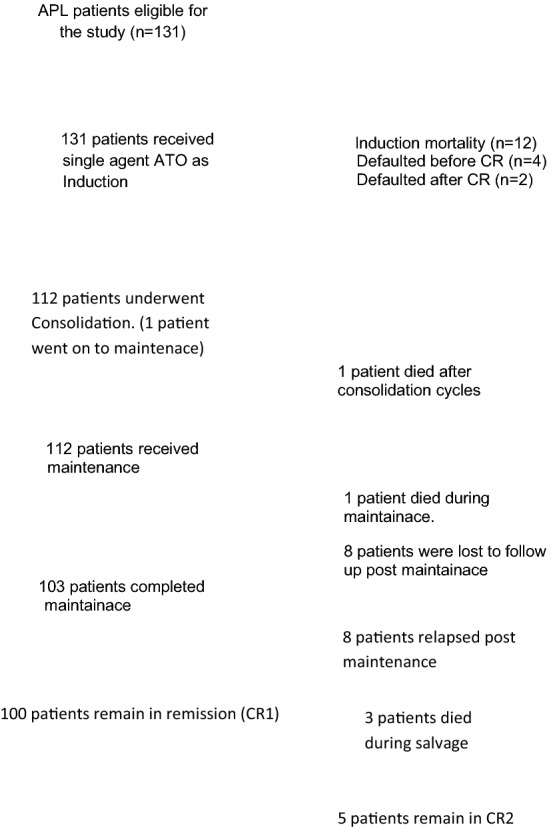
Consort diagram

A total of 131 consecutive patients registered at our centre between January 2009 to December 2012 were included in the analysis. The outcomes are summarized in the consort diagram (Fig. 1). The median age at diagnosis was 30 years (range 14–67 years). Median time from initial diagnosis of APL and evaluation at our center was 3 days (range 0–63 days). About 70% of patients had bleeding manifestations from various sites with 3 patients presenting with CNS bleed, 82% of patients had fever at presentation.

The baseline characteristics were as described in Table 1.

**Table 1:** Baseline characteristics

Baseline parameters	
Sex	Male—N = 72 (55%)
	Female—N = 59 (45%)
	Ratio 1.2:1
Patients over age 60	N = 5 (3.81%)
Total leukocyte count	8900/ml (60–1,31,000)^a^
Platelet count	23,000/ml (4000–2,30,000)^a^
Fibrinogen	310 mg/dl (115–4969)^a^
Sanz risk score	Class I—N-20 (15.3%)
	Class II—N-48 (36.6%)
	Class III—N-63 (48.1%)
Real time qualitative PCR at baseline subtypes (done in 72/131 subjects)	Long transcript—N-48
	Short transcript—N-23
	Variable transcript—N-1
FISH positive for t(15; 17)	N-131

^a^Median (range)

### Induction

All patients were initiated on single agent ATO immediately when a diagnosis of APL was suspected on morphology at 0.15 mg/kg/day (maximum dose-10 mg/day). It was continued once daily until day 45. ATO was temporarily stopped in 36 patients for reasons which included dyselectrolytemia, toxicity and differentiation syndrome (Table 2). The median duration of arsenic stoppage was 3 days following which it was restarted. The median duration for the attainment of complete remission was 51 days.

**Table 2:** ATO Toxicity during induction phase requiring temporary cessation

Arsenic toxicity (n = 38)	Number
QTc prolongation (500 ms)	24
Neuropathy (grade 3/4)	6
Skin desquamation (grade 3/4)	3
Hepatotoxicity (grade 3/4)	3
Hypersensitivity pneumonitis	1
Complete heart block	1

During induction 105 (80.2%) patients received IV antibiotics for variable periods (range 2–60 days). 37 (28.2%) patients also received antifungals. 15 (11.45%) patients required ionotropic support during initial phases of therapy. Differentiation syndrome was seen in 26 (2.7%) of patients. However, injection dexamethasone was used in 47 (35.9%) of patients in anticipation of onset of differentiation syndrome prophylactically, the most common reason being rapidly rising counts. The induction mortality encountered were primarily in the very early phase of therapy, that is within 7 days of starting treatment. 12 out of the 17 total documented deaths were prior to completion of induction. Among 12 induction deaths 8 patients died early due to complications related to sepsis. The remaining 3 early deaths were due to pulmonary/CNS bleed and 1 patient succumbed unrelated pre-existing dilated cardiomyopathy prior to diagnosis of APL. Four patients defaulted, while on induction becoming lost to follow up and were presumed to have died and included as so for the analysis. Following completion of Induction, bone marrow was done in all patients to document remission by morphology, cytogenetics and or molecular studies (Table 3).

**Table 3:** Response evaluation

	Yes	No	Not applicable (death/default)	Not done
Morphologic remission	114 (99.1%)	1 (0.9%)	16	0
Cytogenetic remission	78 (78.78%)	21 (26.9%)	16	16
Molecular remission	52 (65%)	28 (35%)	16	35

### Consolidation

112 patients underwent consolidation, 2 patients defaulted after achieving CR. One patient was directly started on maintenance in view of stormy induction, as she had already achieved molecular remission post induction. She continues to be in molecular remission. The one patient who had not attained morphologic remission, went into CR after the 1st cycle of consolidation. All patients received primary G-CSF prophylaxis. 24 patients developed febrile neutropenia during consolidation with median duration of neutropenia being 4 days (range 1–4). There was no mortality due to chemotherapy during consolidation. One patient died after consolidation, the cause for which could not be ascertained as she died elsewhere. Among the 21 patients who were cytogenetically positivity by FISH for t (15; 17) only 1 remained positive post consolidation. 2 patients persisted to have *PML: RARA* transcript by PCR after consolidation. Molecular CR was documented in 95 (84%) of the evaluable 112 patients, out of which 52 were documented after ATO induction and 43 more after completion of anthracycline plus ATRA based consolidation. 2 patients did not achieve molecular remission, but they were salvaged by further chemotherapy. In 18 (13.75%) patients’ molecular studies were not available due to non-adherence to protocol or non-availability of the results. 16 (12.2%) patients had either died or defaulted before response evaluation could be done.

### Maintenance

Among 112 patients eligible to go in for maintenance and all but 3 received ATRA based maintenance, according to protocol. Three patients received 6MP + MTX + ATRA, in view of suboptimal consolidation therapy received. There was no grade III or IV hematologic or non-hematological toxicity encountered during maintenance phase. However, one patient on 6MP + MTX died due to sepsis during 1st maintenance. 8 patients defaulted and were lost to follow up during or after maintenance.

### Outcomes

**Fig. 2:**
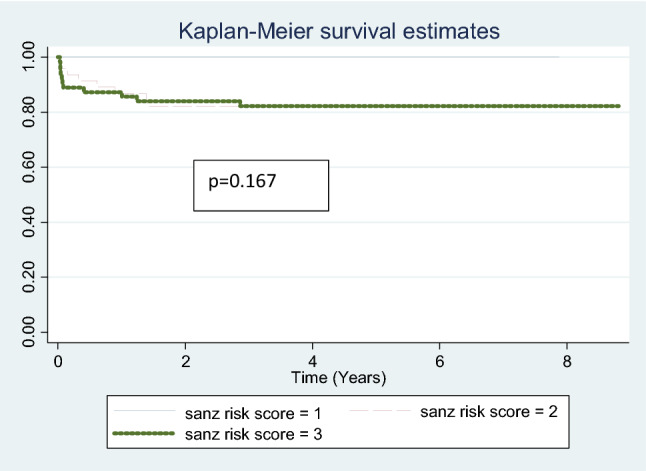
Kaplan Meir curve event free survival stratified for Sanz risk groups

**Fig. 3:**
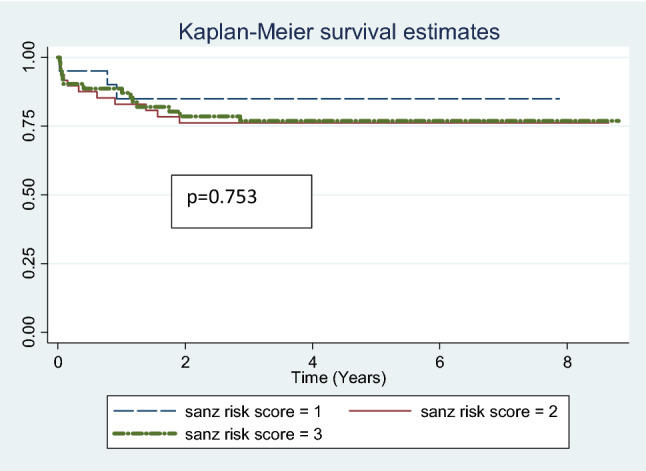
Overall survival stratified for Sanz risk groups

Eight patients relapsed after completion or while on maintenance therapy. One of the eight had a relapse with AML M5a. She was put on palliative care. Five of the remaining seven patients were salvaged with chemotherapy. Two of the seven died during salvage. Among the five patients who were salvaged two have been lost to follow up. At a median follow up of 60 months, (range 36–106 months) the overall event free survival was 77.82%. When stratified by Sanz risk score the event free survival for low risk group was 85% and 76% for intermediate and high-risk groups (Fig. 1). There was no statistically significant difference between the groups (*p* = 0.167). Overall survival was 84.81%. When stratified by Sanz risk score the overall survival for low risk group was 100% and 82% for intermediate and high-risk groups. There was no statistically significant difference in outcomes between low, intermediate and high risk when the sequential approach for treatment was implemented (*p* = 0.753) (Figs. 2, 3).

## Discussion

Acute promyelocytic leukemia (APL) has emerged from a disease with high early mortality to being the most curable form of leukemia with the incorporation of differentiating agents to chemotherapeutic strategies. This has also been made feasible through a clear understanding of the biology of the disease and how targets when perturbed effectively translate into curative outcomes. The treatment of APL over the past 2 decades has evolved from the use of chemotherapy alone to use of differentiating agent with chemotherapy, starting with ATRA and thereafter Arsenic to using combination of differentiating agents with chemotherapy and more recently without for the low and intermediate risk APL. The former strategy remained a standard yielding remission rates of 95% and cure exceeding 80% in various studies [6–10]. The demonstration of activity of ATO in relapsed APL and its ability to induce durable remissions in newly diagnosed APL as single agent, particularly in low risk challenged this dogma [3, 4]. The combination of ATRA and ATO in newly diagnosed APL has since demonstrated superior responses (97% EFS at 2 years as compared with 86% in the ATRA plus chemotherapy arm) and the ability to sustain remission in the low and intermediate risk group APL [5].

The difficult problem that is confronted when treating newly diagnosed APL is early mortality encountered due to bleeding diathesis, infection and chemotherapy induced toxicity. Outside of clinical trials the SEER data and Swedish data revealed an early mortality rate of 17.3% and 26% respectively with patients older than 55 years having a higher early mortality [11, 12]. While reports from Asia have shown early death rates of as high as 42%, published data from India put early mortality at 24.3% [13, 14]. More recently data from Brazil showed early mortality of 20% from a single institution study [15]. Data from use of ATO as a single agent in newly diagnosed APL showed a significantly lower early mortality rate of 9.2% [3, 16]. The need to decrease early mortality in our patient diagnosed with APL among all risk groups was the rationale behind implementation of the use of ATO as single agent for treatment of all newly diagnosed APL. The regimen was well tolerated with no increased toxicity relating to ATO in the form of QTc prolongation (20%), transaminitis (3/118 patients) or differentiation syndrome (2.7%). This was much lesser than documented in published studies. This strategy also achieved its primary objective of decreasing early mortality to 12.1% which was still lower at 9.1% when considering only the patients who were initiated and received induction therapy.

The rationale for embarking on use of ATO as primary therapy irrespective of risk group was based on data that single agent ATO is efficacious in maintaining long term remission status in low risk patient. Besides, as a single agent, it maintained activity in the intermediate and high-risk patient with APL [3]. However, this strategy was also tempered with the knowledge that the intermediate and high-risk APL are more likely to relapse without some form of consolidation in addition to ATO alone. Therefore, we used a treatment strategy with ATRA and chemotherapy as a sequential consolidation phase, an approach never considered in other studies and defined by our group as a sequential therapy. This sequential therapy was not only well tolerated but also administered as an outpatient with minimal toxicity encountered. The strategy was successfully implemented in practically all patients who received the planned therapy. All patients were alive after they completed sequential therapy with only 8 relapses occurring on follow up subsequently. There was no cardiac toxicity documented in any of our patients during or after therapy or on follow up and was irrespective of the dose of anthracyclines (60 mg/m^2^ or 45 mg/m^2^) used. There was no evident compromise in the outcomes either.

The cytogenetic and molecular remission rates (in those analyzed) following induction was 78.7% and 65% respectively indicating the profound single agent activity of ATO in frontline setting. The molecular remission further improved to 84% following consolidation phase. Except for one patient all achieved cytogenetic remission following consolidation documented by fluorescent in situ hybridization. All but three patients received maintenance therapy with ATRA. Patient who did not attain a molecular remission was further consolidated with high dose cytarabine.

The cohort of patient in this study had 48% having high-risk features prone for early mortality and relapse. At a median follow up of 60 months 84% were alive with an EFS of 77.8%. Outside of a clinical trial these outcomes are comparable or even better than those available from population-based data wherein long-term outcomes was only 61% and as low as 59% with a similar duration of follow up in all comers [12, 15]. If one-compared outcomes for patient in the low risk and intermediate/high risk group the EFS was 85% and 76% with overall survival of 100% and 82% respectively. This was again comparable to published studies outside the purview of clinical trial [17].

The outcomes for use of ATRA and ATO in combination, for low and intermediate risk APL, has shown superiority to standard ATRA plus Chemotherapy alone [5]. Therefore, it may be argued that use of this sequential strategy for treating these groups would be an unnecessary overtreatment and hence we have switched over to single agent ATO as described by Lococo et al. in low and intermediate risk APL [5]. However, the long-term data of excellent cure especially in the high-risk group gives credence to this strategy. The relative shorter duration (18 weeks) of implementation as against the 10 months required for the chemotherapy free protocol makes this an effective alternate to patients with all risk group APL especially when follow up can be a challenge in developing countries [18].The maintenance strategy with single agent ATRA was well tolerated and gave the treating team an opportunity to ensure follow-up and compliance. The effectiveness in achieving early cytogenetic and molecular responses which are durable and translates into cure also gives credibility to this approach. The sequential approach for high risk APL could be a recommendation given our long-term data of 6 years post therapy demonstrating lower early mortality with high cures.

To conclude the sequential approach to treating APL stemmed from a need for early control while decreasing mortality which was achieved. The data provides substance for this approach to achieve early responses which also translated into durable remission and cure. The impact on intermediate and high-risk disease would suggest that this strategy may be implemented with conviction as a short-term treatment with low toxicity, low mortality and high cure especially when compliance to long duration treatment may pose a challenge in developing countries.
